# Peripancreatic adipose tissue protects against high-fat-diet-induced hepatic steatosis and insulin resistance in mice

**DOI:** 10.1038/s41366-020-00657-6

**Published:** 2020-08-25

**Authors:** Belén Chanclón, Yanling Wu, Milica Vujičić, Marco Bauzá-Thorbrügge, Elin Banke, Peter Micallef, Julia Kanerva, Björn Wilder, Patrik Rorsman, Ingrid Wernstedt Asterholm

**Affiliations:** 1 Department of Physiology (Metabolic Physiology Research Unit), Institute of Neuroscience and Physiology, Sahlgrenska Academy, University of Gothenburg, Box 432, SE405 30 Gothenburg, Sweden; 2 Oxford Centre for Diabetes, Endocrinology and Metabolism, Radcliffe Department of Medicine, University of Oxford, Oxford, OX4 7LE UK

**Keywords:** Obesity, Medical research

## Abstract

**Background/objectives:**

Visceral adiposity is associated with increased diabetes risk, while expansion of subcutaneous adipose tissue may be protective. However, the visceral compartment contains different fat depots. Peripancreatic adipose tissue (PAT) is an understudied visceral fat depot. Here, we aimed to define PAT functionality in lean and high-fat-diet (HFD)-induced obese mice.

**Subjects/methods:**

Four adipose tissue depots (inguinal, mesenteric, gonadal, and peripancreatic adipose tissue) from chow- and HFD-fed male mice were compared with respect to adipocyte size (*n* = 4–5/group), cellular composition (FACS analysis, *n* = 5–6/group), lipogenesis and lipolysis (*n* = 3/group), and gene expression (*n* = 6–10/group). Radioactive tracers were used to compare lipid and glucose metabolism between these four fat depots in vivo (*n* = 5–11/group). To determine the role of PAT in obesity-associated metabolic disturbances, PAT was surgically removed prior to challenging the mice with HFD. PAT-ectomized mice were compared to sham controls with respect to glucose tolerance, basal and glucose-stimulated insulin levels, hepatic and pancreatic steatosis, and gene expression (*n* = 8–10/group).

**Results:**

We found that PAT is a tiny fat depot (~0.2% of the total fat mass) containing relatively small adipocytes and many “non-adipocytes” such as leukocytes and fibroblasts. PAT was distinguished from the other fat depots by increased glucose uptake and increased fatty acid oxidation in both lean and obese mice. Moreover, PAT was the only fat depot where the tissue weight correlated positively with liver weight in obese mice (*R* = 0.65; *p* = 0.009). Surgical removal of PAT followed by 16-week HFD feeding was associated with aggravated hepatic steatosis (*p* = 0.008) and higher basal (*p* < 0.05) and glucose-stimulated insulin levels (*p* < 0.01). PAT removal also led to enlarged pancreatic islets and increased pancreatic expression of markers of glucose-stimulated insulin secretion and islet development (*p* < 0.05).

**Conclusions:**

PAT is a small metabolically highly active fat depot that plays a previously unrecognized role in the pathogenesis of hepatic steatosis and insulin resistance in advanced obesity.

## Introduction

Proper storage of excess nutrients in adipose tissue during weight gain protects against the deleterious ectopic lipid deposition in non-adipose tissues. Typically, mouse models with a high capacity for hyperplastic subcutaneous adipose tissue expansion are protected against ectopic lipid deposition and its associated metabolic disturbances [1, 2]. In contrast, increased visceral adiposity is a predictor of metabolic syndrome and insulin resistance [3–7]. Consequently, subcutaneous adipose tissue is regarded as “good fat” while visceral adipose tissue as “bad fat.” In line with this assumption, partial lipectomy of visceral fat improved the metabolic profile [3], and transplantation of gonadal fat accelerated atherogenesis in apolipoprotein E-deficient mice, whereas subcutaneous fat transplantation had no effect [8].

One possible explanation for the association between visceral adiposity and increased disease risk is that visceral adipose tissue is more prone to become chronically inflamed than subcutaneous adipose tissue [3, 4, 9]. However, there are also regional differences with respect to lipolysis [10], fatty acid storage [11], adipokine secretion [6], and overall gene expression profile [12, 13]. Thus, there are intrinsic metabolic and immunological differences between visceral and subcutaneous adipose tissue.

Peripancreatic adipose tissue (PAT) is a visceral fat depot that should not be confused with intrapancreatic adipocytes, which have received significant attention the past 15 years (as reviewed in ref. [14]). Both PAT and intrapancreatic adipocytes are implicated in the development of diabetes, but whether peripancreatic and intrapancreatic adipocytes display similar function and/or stem from similar precursor cells are currently unknown. Intrapancreatic adipocytes appear to arise within pancreas primarily when conditions are sufficiently adipogenic such as in response to high-fat diet (HFD) feeding in mice [15–17]. The amount of intrapancreatic adipocytes correlates with the pancreatic fat content and have been suggested to protect the pancreas against ectopic lipid deposition, but these adipocytes could also affect islet functionality positively or negatively through their release of adipokines and fatty acids [15–18]. In contrast, PAT is a distinct fat depot containing both adipocytes and “non-adipocytes”. To date, only a few studies have focused on PAT, but evidence for cross talk between PAT and beta cells during the development of obesity has been provided from a combination of transcriptomics, proteomics, and metabolomics of PAT and islets in rats [19]. It has also been shown that obesity- and diabetes-induced changes in PAT, but not gonadal (GWAT) and inguinal white adipose tissues (IWAT), secretome increase beta-cell proliferation in vitro [20, 21]. Moreover, mouse and human PAT depots have also been shown to contain inflammatory foci in the form of lymphoid-like structures [22, 23], and pro-inflammatory activity of PAT has been suggested to be responsible for the HFD/obesity-induced acceleration of pancreatic neoplasia in a mouse model of pancreatic cancer [23]. Nevertheless, the intrinsic characteristics of PAT and its potential role in diabetes are still largely unknown. Here, we have performed a morphological and functional study of PAT in comparison with the more commonly studied mesenteric (M)WAT, GWAT, and IWAT in mice. We demonstrate that PAT constitutes a metabolically active fat depot that contains small adipocytes and a relatively large amount of stromal vascular cells. Furthermore, surgical removal of PAT aggravated hepatic steatosis and hyperinsulinemia in HFD-induced obesity.

## Material and methods

### Animals and design of diet-induced obesity study

All animal experiments were approved by the Animal Ethics Committee at the Administrative Court of Appeals in Gothenburg, Sweden. Adult male *C57Bl/6J* wild-type mice (Charles River, Sulzfeld, Germany) were maintained with ad libitum access to water and standard chow diet as previously described [24]. In the diet-induced obesity time course study, mice were randomly assigned to groups and fed standard chow or HFD (60% fat, 20% protein, and 20% carbohydrate, D12492, Research Diets Inc., New Brunswick, NJ, USA) the last 1, 4, 8, and 16 weeks before tissue collection at 16 weeks of age (except the 16-week chow and HFD groups, which were sacrificed at 24 weeks of age). All animals were fasted for 4 h prior tissue collection. Trunk blood (serum) was collected, GWAT (unilaterally), IWAT (unilaterally), MWAT, PAT, pancreas, and liver were weighed and either snap-frozen in liquid nitrogen and stored at −80 °C until analysis or collected for histological analysis. Lipid and glucose uptake studies, flow cytometry, quantiative real-time PCR, perilipin-1 staining, and histological methods are described in Supplementary methods.

### Surgical removal of PAT

**Fig. 1:**
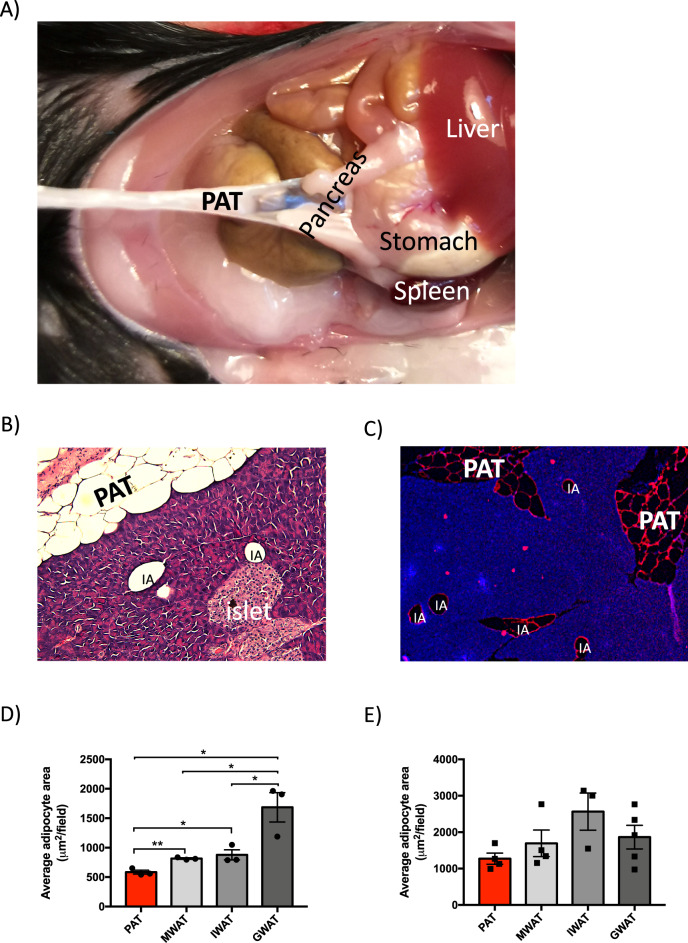
Anatomical and morphological characterization of PAT in chow-diet-fed male mice. **a** Anatomical location of mouse PAT. Representative **b** H&E and **c** perilipin-1 (red) stained sections of mouse pancreas including PAT versus intrapancreatic adipocytes (IA). Adipocyte size of PAT, MWAT, IWAT and GWAT in **d** chow-diet-fed mice (*n* = 7–10 mice) and **e** 8-week HFD-fed mice (*n* = 4–5 mice). All values are expressed as mean ± SEM. *p < 0.05 and **p < 0.01 for the indicated comparisons. PAT peripancreatic adipose tissue, MWAT mesenteric adipose tissue, IWAT inguinal adipose tissue, and GWAT gonadal adipose tissue.

Littermate male *C57Bl/6J* mice (8–10 weeks of age) were randomly divided into control or PAT-ectomy groups, anaesthetized with isoflurane (Apoteksbolaget, Göteborg, Sweden, 2.0% air mixture) and placed on a heated pad to reduce heat loss. The mice were incised in their left side of abdomen and PAT (5 ± 0.4 mg) was identified in the peritoneal cavity using spleen as a landmark (Fig. 1a). PAT was carefully resected in the PAT-ectomy group while control mice underwent sham surgery where PAT was cut but not removed. Therafter the peritoneum was sutured (V489H, resorbable polyglactin 910, Coated VICRYL Ethicon Inc, Scotland), the skin stapled (Reflex 7-mm clip system, Agntho’s, Sweden), and to relieve postoperative pain Temgesic (0.05 mg/kg; Apoteksbolaget) was given intraperitoneally. Prior to being returned to home cages, the mice were allowed to recover on a heated pad. Oral glucose tolerance test, tissue triglyceride quantification, islet and PAT co-culture, and glucose-stimulated insulin secretion are described in Supplementary methods.

### Statistical analyses

GraphPad Prism 7 (GraphPad Software, San Diego, CA, USA) was used for statistical analysis. The sample sizes in each experiment are provided in results text and in figure legends. Only ill and/or wounded animals were excluded from the analyses (*n* = 3 in this study). Data are presented as mean ± SEM. Comparisons were performed using one-way or two-way ANOVA or two-tailed Student’s *t* test. The variance was similar between compared groups. Pearson *r* correlation was used to measure the degree of the relationship between adipose tissue and liver weights. Data were log-transformed as necessary to achieve normal distributions and *p* < 0.05 was considered significant.

## Results

### PAT contains smaller adipocytes than MWAT, IWAT, and GWAT

PAT is an independent fat depot, distinct from intrapancreatic adipocytes, that is located in the ventromedial area attached to the pancreas (Fig. 1a–c). In healthy chow-fed mice PAT weighed 5–10 mg, ~0.2% of the total fat mass based on DEXA data [25]. We found that PAT adipocytes were on average smaller than MWAT, IWAT, and GWAT adipocytes (Fig. 1d). Also, the maximal adipocyte size was smaller in PAT than in the other fat pads (Supplementary Fig. 1A). These differences disappeared following 8-week HFD feeding (Fig. 1e and Supplementary Fig. 1B).

### PAT displays an altered cellular composition compared to other fat depots

**Fig. 2:**
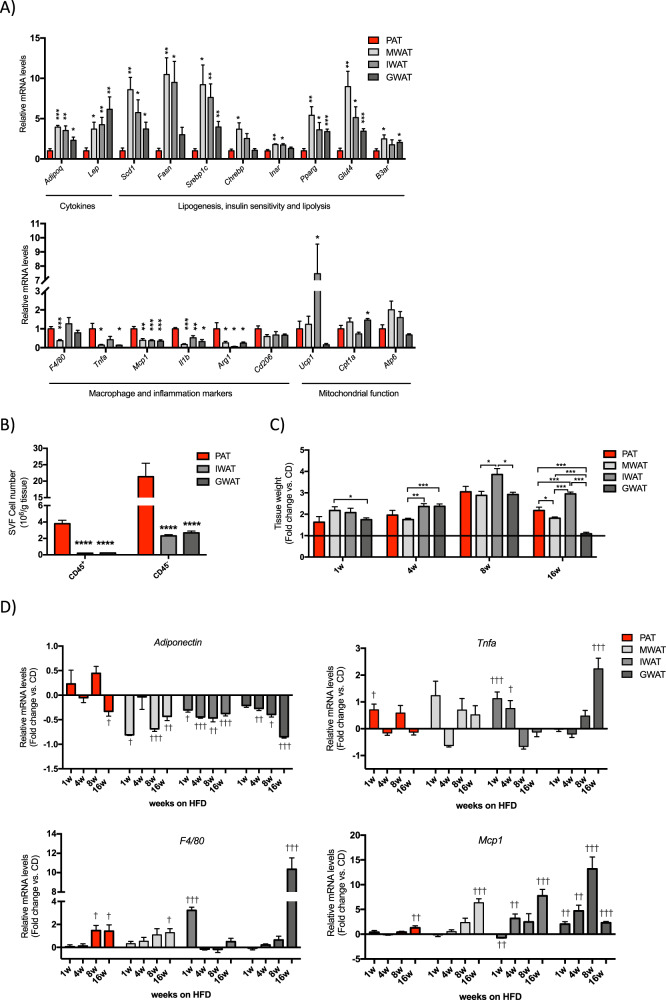
Gene expression profile assessed by quantitative real-time PCR in PAT, MWAT, IWAT, and GWAT. **a** Relative mRNA levels in PAT, MWAT, IWAT, and GWAT on chow-diet conditions (normalized to *Actb*). Data are expressed relative to the level in PAT. **b** FACS analysis of CD45^+^ (leukocytes) and CD45^−^ cells in SVF isolated from PAT, IWAT, and GWAT. **c** Tissue weights of PAT, MWAT, IWAT, and GWAT in mice after 1-, 4-, 8- and 16-week HFD feeding, represented as fold-change from chow-diet-fed aged matched controls. **d** Relative mRNA levels in PAT, MWAT, IWAT, and GWAT in mice after 1-, 4-, 8- and 16-week HFD feeding (normalized to *Actb* and *Tbp*), represented as fold-change from chow diet. All values are expressed as mean ± SEM (*n* = 6 mice/group in 1, 4, and 8 weeks on HFD vs. CD; *n* = 10 mice/group in 16 weeks on HFD vs. CD). *p < 0.05, **p < 0.01, and ***p < 0.001 for PAT vs. other depots; †*p* < 0.05, ††*p* < 0.01, and †††*p* < 0.001 for mice-fed high-fat diet vs. chow diet. *Adipoq* adiponectin, *Lep* leptin, *Scd1* stearoyl-coenzyme A desaturase 1, *Fasn* fatty acid synthase, *Srebp1c* sterol regulatory element-binding transcription factor-1c, *Chrebp* carbohydrate-responsive element-binding protein, *Insr* insulin receptor, *Pparg* peroxisome proliferator-activated receptor gamma, *Glut4* glucose transporter type 4, *B3ar* adrenoceptor beta 3, *Tnfa* tumor necrosis factor alpha, *Mcp1* monocyte chemoattractant protein-1, *Il1b* interleukin-1 beta, *Arg1* arginase 1, *Cd206* macrophage mannose receptor C-type 1, *Ucp1* uncoupling protein-1, *Cpt1b* carnitine palmitoyltransferase 1b, *Atp6* ATP synthase 6, PAT peripancreatic adipose tissue, MWAT mesenteric adipose tissue, IWAT inguinal adipose tissue, and GWAT gonadal adipose tissue.

PAT displayed a different gene expression profile compared to MWAT, IWAT, and GWAT. The mRNA levels of genes encoding leptin, adiponectin, markers of lipogenesis, insulin sensitivity, and lipolysis were significantly lower in PAT than in MWAT, IWAT, and GWAT. Conversely, the expression of many inflammatory markers was higher in PAT. PAT also displayed higher expression of the M2-type macrophage marker arginase 1 and a similar trend was seen for the macrophage mannose receptor C-type 1 (*Cd206*). *Atp6* and other mitochondrial markers levels were similar between depots except for a higher expression of *Ucp1* and *Cpt1a* in IWAT and GWAT, respectively (Fig. 2a). Despite this gene expression pattern, PAT adipocytes appeared just as functional as MWAT adipocytes judged by basal and insulin-stimulated de novo lipogenesis (Supplementary Fig. 1C). Lipolysis, induced by β_3_-adrenergic receptor agonist CL-316,243 (CL), was greater in PAT adipocyte while there was a trend toward decreased basal lipolysis in PAT compared to MWAT adipocytes (Supplementary Fig. 1D). Based on these data, we hypothesized that the altered gene expression pattern reflects a different cellular composition (with fewer adipocytes and more leukocytes in PAT) rather than an elevated pro-inflammatory dysfunctional state. Indeed, PAT displayed ten times more stromal vascular cells per g of tissue compared to IWAT or GWAT (Fig. 2b). Furthermore, PAT contained more fibroblasts, more macrophages, more dendritic cells, and more lymphocytes than IWAT and GWAT (Supplementary Fig. 1E).

### PAT is protected against some of the HFD-induced transcriptional changes in adipose tissue

Body weight in HFD-fed mice increased by 13%, 22%, 52%, and 54% after 1, 4, 8, and 16 weeks, respectively, compared to age-matched chow-fed control mice (Supplementary Fig. 2A, B). The relative fat pad weight increase compared to chow controls was similar between PAT and the other fat depots for the first 8 weeks on HFD (Fig. 2c). After 16-week HFD feeding, PAT showed a higher relative weight increase compare to MWAT (*p* = 0.03) and GWAT (*p* < 0.001), although IWAT gained most weight of the four fat depots (*p* < 0.001) (Fig. 2c). The relative difference in fat depot weight between HFD- and chow-fed mice at 16 weeks was reduced compared to the 8-week HFD group. The reason for this relative reduction is due to significant fat mass gain also in the ad libitum chow-fed controls (Supplementary Table 1). Despite similar relative fat depot weight gain during the first 8 weeks on HFD, the gene expression pattern was different in PAT compared to the other fat depots. In PAT, the adiponectin mRNA levels were reduced only after 16 weeks on HFD, while the expression of these genes was reduced already after 1-week HFD feeding in the other fat depots (Fig. 2d). The pro-inflammatory cytokine *Tnfa* increased slightly after 1-week HFD feeding in PAT and IWAT, while GWAT displayed a marked increase after 16 weeks on HFD (Fig. 2d). The PAT and MWAT mRNA levels of the pan-macrophage marker *F4/80* increased with duration of HFD. For IWAT and GWAT, the *F4/80* responses were more variable: large increases were observed after 1 week (IWAT) and 16 weeks (GWAT), but not at other times (Fig. 2d). The monocyte chemoattractant *Mcp1* was modestly regulated in PAT with a significant increase only after 16 weeks on HFD. A similar pattern was seen in MWAT although the HFD-induced upregulation at 16 weeks was higher than in PAT. In contrast, GWAT *Mcp1* levels were upregulated throughout the HFD-time course and highest after 8-week HFD feeding, and IWAT *Mcp1* levels were reduced after 1 week on HFD followed by an upregulation after 4 and 16 weeks on HFD (Fig. 2d).

### PAT has a higher capacity for lipid oxidation and glucose uptake than MWAT, IWAT, and GWAT

**Fig. 3:**
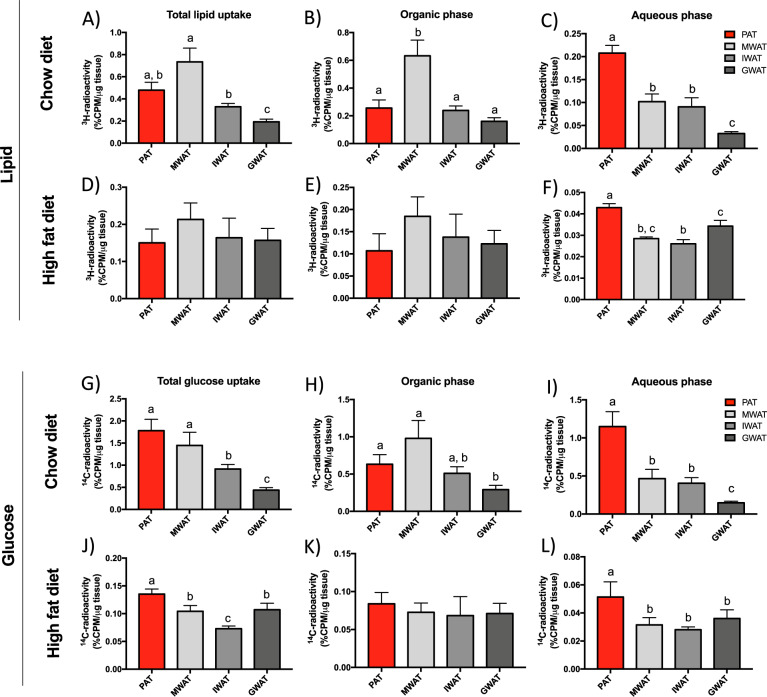
Lipid and glucose metabolism in PAT, MWAT, IWAT, and GWAT in chow- and 16-week HFD-fed male mice. **a, d** Total lipid uptake, **b, e** triglyceride accumulation (organic phase), and **c, f** lipid oxidation (aqueous phase) in different fat depots in respectively, chow (*n* = 8–11 mice/group) and 16-week HFD-fed mice (*n* = 5–6 mice/group). **g, j** Total glucose uptake, **h, k** de novo lipogenesis from glucose (organic phase), and **i, l** glucose catabolism (aqueous phase) in respectively, chow (*n* = 8 mice/group) and 16-week HFD-fed mice (*n* = 5–6 mice/group). All values are expressed as mean ± SEM. Values that do not share a common letter (a, b, and c) are statistically different. PAT peripancreatic adipose tissue, MWAT mesenteric adipose tissue, IWAT inguinal adipose tissue, and GWAT gonadal adipose tissue.

In chow-fed mice, total lipid uptake (^3^H-triolein counts per µg tissue) varied between the different depots: highest in MWAT and lowest in GWAT with PAT being intermediate (Fig. 3a). Triglyceride accumulation (judged by the ^3^H counts in the organic phase) was greatest in MWAT and ~2.5-fold higher than in the other depots (Fig. 3b). Interestingly, the depot with the highest lipid oxidation (judged by ^3^H counts in the aqueous phase) was PAT where 43% of the total uptake was found in the aqueous phase compared to the 14%, 27%, and 16% in MWAT, IWAT, and GWAT, respectively (Fig. 3c). In mice-fed HFD for 16 weeks, the total lipid uptake and triglyceride accumulation were similar between depots (Fig. 3d, e). However, lipid oxidation was again highest in PAT (Fig. 3f).

In chow-fed mice, total glucose uptake in PAT and MWAT was similar, and higher than in IWAT and GWAT (Fig. 3g). In obese, HFD-fed conditions, PAT showed the highest glucose uptake: 30%, 85%, and 26% greater than MWAT, IWAT, and GWAT, respectively (Fig. 3j). Lipogenesis (estimated from ^14^C counts in the organic phase) was highest in MWAT and lowest in GWAT in chow-fed mice (Fig. 3h), whereas levels were similar between all fat depots in HFD-fed mice (Fig. 3k). For the aqueous phase (at least in part reflecting glucose oxidation), the highest ^14^C counts were found in PAT where levels were tenfold higher than in GWAT in mice on chow (Fig. 3i). In HFD-fed mice, the highest ^14^C counts in the aqueous phase were also found in PAT with levels in the other depots being about half (Fig. 3l). Collectively, these data suggest that PAT is a metabolically active depot with a relatively high capacity for lipid and glucose oxidation.

### PAT weight is positively correlated with liver weight in HFD-induced obesity

**Fig. 4:**
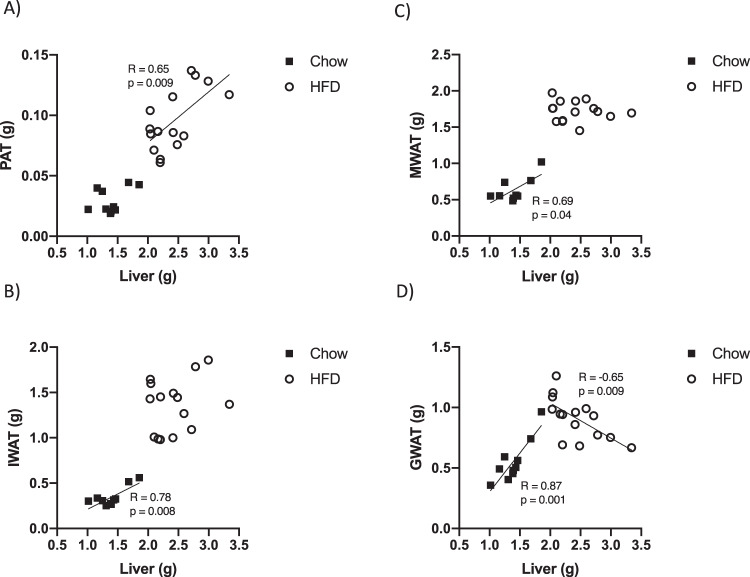
Pearson correlations between different fat depots and liver weight in chow and 16-week HFD-fed male mice. Pearson correlation was used to measure the degree of correlation between liver weight and, respectively, **a** PAT weight, **b** IWAT weight, **c** MWAT weight, and **d** GWAT weight. Black squares indicate mice fed with chow diet and white circles indicate mice fed with HFD. A *p* value < 0.05 indicates that the Pearson’s correlations coefficient (*R*) is significantly different from zero. PAT peripancreatic adipose tissue, MWAT mesenteric adipose tissue, IWAT inguinal adipose tissue, and GWAT gonadal adipose tissue.

We performed correlation analyses of body, adipose tissue, and liver weights. In lean chow-fed mice, there was a positive correlation between body weight and the weight of all fat depots. There was also a positive correlation between body and liver weights (Supplementary Table 2). Following 16 weeks on HFD, there was a positive correlation between IWAT and liver weight and a similar trend was seen for PAT (Supplementary Table 2). In contrast, there was no correlation between body weight and GWAT and body weight and MWAT, possibly indicating that these depots have reached their maximal size and cannot expand further (Supplementary Table 2). It has been reported that there is a significant amount of adipocyte death in GWAT after chronic HFD feeding [26], which may explain the paradoxical relative reduction in GWAT size in advanced obesity in mice. In chow-fed mice, there was a positive correlation between liver weight and the weight of MWAT, IWAT, and GWAT (Fig. 4b–d; black symbols; *p* = 0.04, *p* = 0.008, *p* = 0.001, respectively). A similar trend, albeit not statistically significant, was observed for PAT (Fig. 4a). In HFD-fed mice, only PAT weight showed a clear significant correlation with liver weight (Fig. 4a; white circles; *p* = 0.009). For the other depots, no such correlation was observed and for GWAT a negative correlation was observed (Fig. 4b–d).

### Surgical removal of PAT aggravates HFD-induced hepatic steatosis

Healthy adipose tissue expansion protects against ectopic lipid deposition in the liver [1]. Consequently, hepatic steatosis suggests that excess calories are not efficiently stored in adipose tissue, possibly reflecting adipocyte insulin resistance and inability to expand the fat depots. Thus, we reasoned that the observed positive correlation between PAT and liver weight in advanced obesity (that was not seen for the other three fat depots) suggests that PAT can continue to store excess calories when IWAT, MWAT, and GWAT have reached their limit for healthy expansion. To test this we studied the impact of PAT removal on metabolic function and hepatic steatosis (Supplementary Fig. 3A). Control mice underwent sham surgery. The mice tolerated the surgery well and there was no difference in HFD-induced body-weight gain between the groups. PAT weight in PAT-ectomized mice was only 18% of that in controls after 16-week HFD feeding (103 ± 11 mg vs. 19 ± 5 mg, *p* < 0.001). Thus, our surgical procedure led to a significant difference in the resultant PAT size in HFD-challenged mice although PAT to some extent regenerated over time. The average PAT adipocyte size was similar between groups (6547 ± 853 μm^2^ in controls vs. 6299 ± 430 μm^2^ in PAT-ectomized mice, *n* = 4 + 5, *p* = 0.79). However, there was a difference in size distribution with fewer adipocytes of the smallest size and more adipocytes of the medium to large size in the regenerated PAT of PAT-ectomized mice (Supplementary Fig. 3B). No compensatory increase of IWAT, MWAT, or GWAT weight was detected in PAT-ectomized mice (Supplementary Fig. 3C).

**Fig. 5:**
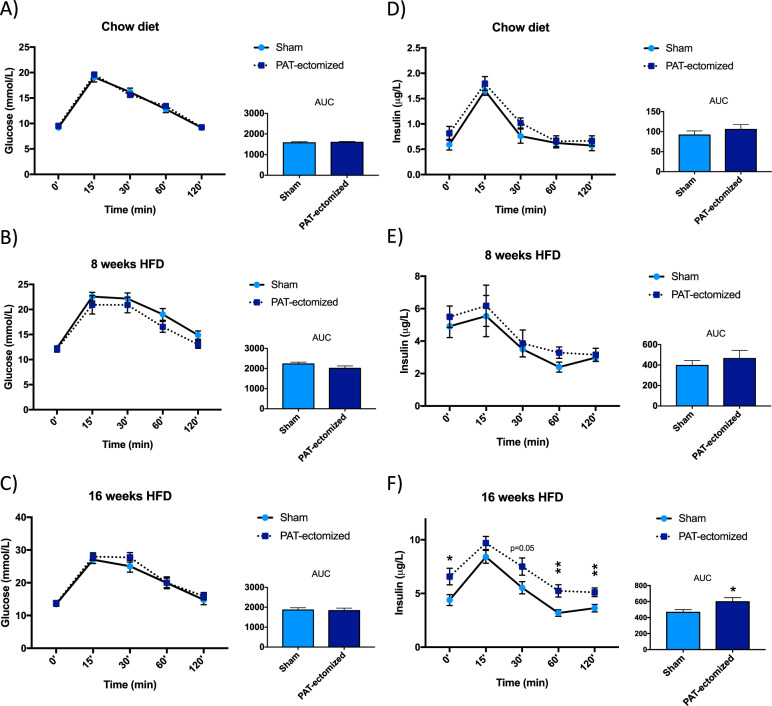
Oral glucose tolerance tests in male PAT-ectomized and sham control mice on chow diet and after 8- and 16-week HFD-feeding. Circulating glucose levels at indicated time points and AUC in response to oral glucose load in PAT-ectomized and sham controls mice on **a** chow diet (CD; *n* = 7–9 mice/group), after **b** 8 weeks on HFD (*n* = 8–10 mice/group), and **c** 16 weeks on HFD (*n* = 8–10 mice/group). Circulating insulin levels at indicated time points and AUC in response to oral glucose load in PAT-ectomized and sham controls mice on **d** chow diet, after **e** 8 weeks on HFD, and **f** 16 weeks on HFD. All values are expressed as mean ± SEM. *p < 0.05.

Oral glucose tolerance tests performed on mice fed chow and after 8-week HFD feeding revealed no differences between control and PAT-ectomized mice (Fig. 5a, b, d, e). As expected, serum insulin levels were greater than fivefold higher in HFD- than chow-fed mice (reflecting increased insulin resistance). After 16 weeks on HFD, PAT-ectomized mice displayed increased insulin levels (basal as well as after a glucose challenge) despite blood glucose and glucagon levels not being affected (Fig. 5c, f and glucagon levels of 10.0 ± 2.3 in controls (*n* = 7) and 7.2 ± 1.3 pg/mL in PAT-ectomized mice (*n* = 10), *p* = 0.27).

**Fig. 6:**
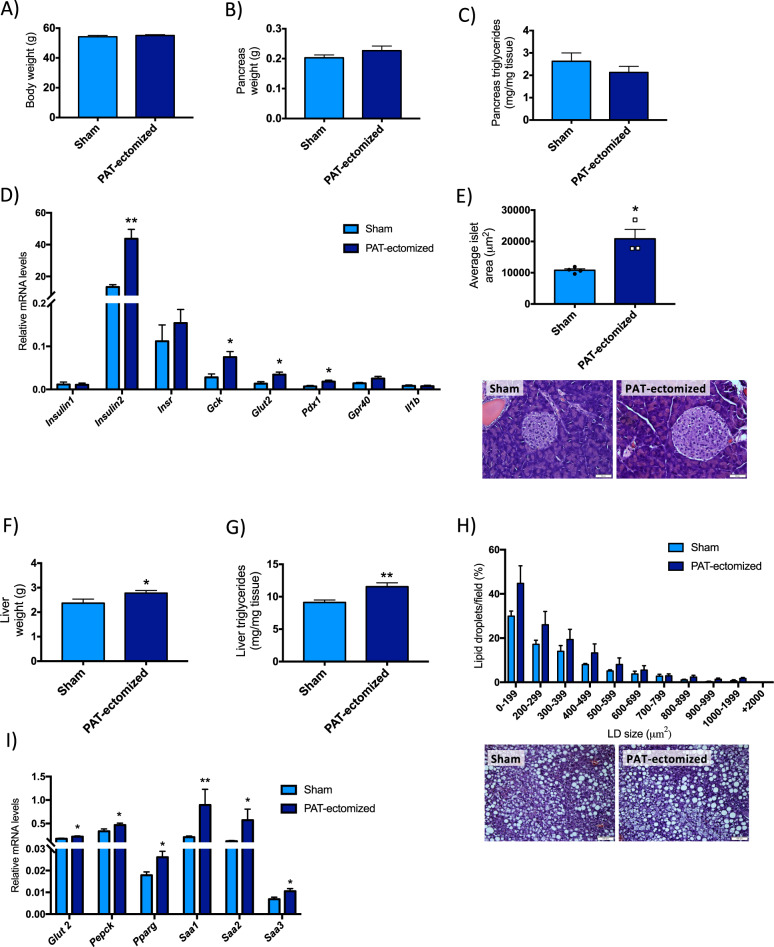
Effect of PAT-ectomy on body weight and, hepatic and pancreatic ectopic lipid deposition and gene expression after 16-week HFD feeding in male mice. **a** Body weight, **b** pancreas weight, **c** pancreatic triglyceride content, and **d** pancreatic gene expression in PAT-ectomized and sham control mice (*n* = 8–10 mice/group). **e** Average pancreatic islet area in PAT-ectomized and sham control mice and representative H&E sections (*n* = 3–4 mice/group). **f** Liver weight, **g** hepatic triglyceride content, **h** hepatic lipid droplet distribution and representative H&E section (*n* = 4–6 mice/group), and **i** hepatic gene expression in PAT-ectomized and sham control mice (*n* = 8–10 mice/group). All values are expressed as mean ± SEM. *p < 0.05 and **p < 0.01.

Body weight (Fig. 6a), pancreas weight (Fig. 6b) and its triglyceride content (Fig. 6c) did not significantly differ between control and PAT-ectomized mice. There were however weak trends toward reduced pancreatic triglyceride content (Fig. 6c, *p* = 0.29) and reduced number of intrapancreatic adipocytes (*p* = 0.17) in PAT-ectomized mice (23.6 ± 4 adipocytes of an average size of 7649 ± 625 μm^2^ in controls vs. 14.8 ± 4 adipocytes of an average size of 7279 ± 710 μm^2^ in PAT-ectomized mice, *n* = 4 + 3). Moreover, pancreatic mRNA levels of insulin (*Ins2*), glucokinase (*Gck*), glucose transporter 2 (Glut2, encoded by *Slc2a2*), and the pancreatic and duodenal homeobox 1 (*Pdx1*) were higher in PAT-ectomized than in control mice (Fig. 6d). In accordance with this gene expression pattern, the average islet area was significantly larger in PAT-ectomized mice, while the number of islets per field were similar between groups (Fig. 6e and 24.9 ± 4 islets in controls vs. 26.5 ± 5 islets in PAT-ectomized mice, *n* = 4 + 3). Furthermore, PAT-ectomized mice displayed more severe HFD-induced hepatic steatosis than controls; the liver weights were increased (Fig. 6f) along with increased triglyceride content (Fig. 6g). Thus, the total liver triglyceride content was almost 50% higher in PAT-ectomized than in control mice. This increase in hepatic triglyceride levels was associated with larger lipid droplets and a trend toward increased “ballooning” i.e., enlarged cells with rarefied cytoplasm (Fig. 6h and Supplementary Fig. 3D). The hepatic expression of inflammatory markers serum amyloid a1, a2, and a3 (*Saa1–3*), as well as markers of insulin resistance such as *Glut2* (*Slc2a2*), phosphoenolpyruvate carboxylase (*Pepck)*, and peroxisome proliferator-activated receptor gamma (*Pparg)*, were elevated in PAT-ectomized mice compared to controls (Fig. 6i).

### PAT co-culture preserves islet functionality in vitro

The close proximity of PAT to the pancreas suggests it may affect islet function. To test this, we analyzed the effect of PAT co-culture on insulin secretion in isolated islets. We found that 18-h co-culture with PAT at 11-mM glucose slightly (~10%) reduced the insulin levels in the co-culture media (Supplementary Fig. 4A). In contrast, the insulin release in response to high glucose post co-culture was higher in the PAT co-culture group than in the controls. This difference in insulin release after co-culture was due to impaired glucose-stimulated insulin release in the control group (Supplementary Fig. 4B).

## Discussion

Healthy adipose tissue can effectively store excess energy during nutrient overload. This storage capacity is often lost in obese individuals. However, the adverse consequences of obesity are not necessarily related to the total body-fat content, but rather to the fat distribution [6, 7]. In general, subcutaneous fat accumulation provokes less metabolic disturbances than visceral adiposity, which is strongly associated with insulin resistance and type-2 diabetes [5, 7]. Nevertheless, subcutaneous fat expansion may also be pathological; insufficient adipogenic potential in the subcutaneous compartment may result in adipocyte hypertrophy and increased risk for developing type-2 diabetes [27, 28]. The PAT is a small fat depot closely located to MWAT within the visceral compartment, but differs from MWAT as well as from other fat depots, both structurally and functionally. We now show that PAT of unchallenged mice contains smaller adipocytes than the other fat depots, suggestive of a higher capacity for triglyceride storage and better sensitivity to insulin and adrenergic activation (cf. [1]). In keeping with this idea, lipolysis in PAT adipocytes displayed a greater sensitivity to CL than that observed in MWAT adipocytes, while insulin-stimulated lipogenesis was similar between the two depots. Interestingly, the lipid and glucose catabolism ([^3^H]-triolein and [^14^C]-glucose counts in the aqueous phase) was particularly high in PAT, indicating that it is a metabolically very active depot. The gene expression profile and the cellular composition in normal chow-diet mice was also different in PAT compared to the other fat depots. In essence, PAT displayed reduced expression of genes related to adipocyte function while the expression of macrophage and inflammatory markers was increased along with a higher number of leukocytes (including macrophages) per gram of tissue. Our gene expression and FACS data are also well in line with one human and one mouse study where PAT is identified as a highly heterogenous tissue with many different lymphoid-like structure and high immunological activity [22, 23]. In fact, one provocative idea is that the immune response on beta cells in type-1 and type-2 diabetes could be initiated by PAT [22]. Our mouse model will allow this hypothesis to be tested.

PAT also displayed relative resistance to the deleterious effects of HFD and the transcriptional changes were not as pronounced as in the other fat depots. For instance, we found decreased PAT adiponectin expression and increased PAT *Mcp1* expression only after 16 weeks on HFD. The PAT expression of the macrophage marker *F4/80* was however increased both at 8 and 16 weeks on HFD. A similar increase in *F4/80* without upregulated *Mcp1* was seen in IWAT after 1-week HFD feeding. *Mcp1* is a chemokine that typically is involved in adipose tissue macrophage recruitment in obesity/type-2 diabetes [29]. Thus, the increase in PAT and IWAT *F4/80*, respectively, at 8- and 1-week HFD feeding, may reflect another macrophage subtype than those seen in e.g., crown-like structures of adipose tissue in advanced obesity. For instance, an increased number of resident macrophages may be required for the necessary tissue remodeling to accommodate the growing adipocytes (or the adipocyte hyperplasia) during adipose tissue expansion [9, 30]. Moreover, the detected 1-week HFD-induced increase in *Tnfa* in IWAT and PAT is also in line with the idea that a potent acute inflammatory response is essential for healthy adipose tissue expansion [30]. These data suggest that PAT (similar to IWAT) has an intrinsically high capacity for healthy expansion, and start suffering from the deleterious effects of diet-induced obesity at later states than many other fat depots.

Visceral adiposity is highly associated with increased liver fat accumulation [31, 32] and is considered as a marker of dysfunctional subcutaneous adipose tissue. Visceral adiposity has been suggested to actively contribute to ectopic fat deposition and metabolic dysfunction through increased release of free fatty acids into the portal vein and increased production of pro-inflammatory cytokines [33, 34]. Moreover, lipectomy studies show that selective reduction in intra-abdominal adipose tissue can improve the metabolic profile by reversing insulin resistance and glucose intolerance in human obesity [3]. Two clinical studies show that omental fat removal is associated with improved insulin sensitivity [35, 36], but such positive effect of visceral fat removal is not always observed [37, 38]. The variable outcome from visceral fat removal may depend on the functionality of the excised adipose tissue. In order to elucidate whether PAT plays a significant role in obesity-associated metabolic disturbances, we surgically removed PAT in lean mice followed by a 16-week long HFD-feeding regimen. Although PAT regenerated to some small extent, likely due to both hypertrophy of remnant adipocytes and formation of new adipocytes, there was still (after 16-week HFD feeding) a >80% difference in PAT size between groups and we found that PAT-ectomized mice displayed higher basal and glucose-induced insulin levels associated with aggravated hepatic steatosis. Thus, PAT expansion protects against hepatic steatosis rather than being a contributing factor. The enhanced hyperinsulinemia in PAT-ectomized mice did not stimulate increased intrapancreatic adipogenesis. If anything, there was a weak trend toward reduced amount of intrapancreatic adipocytes/fat in PAT-ectomized mice possibly indicating that intrapancreatic adipocytes can arise from precursors that reside within PAT.

PAT has been suggested to play a direct role in the regulation of endocrine pancreas [19–21]. In some support of this idea, PAT co-culture preserved the insulin response to high glucose in isolated islets, implying that PAT can protect islets. Thus, our co-culture data corroborate the report suggesting that PAT releases a factor that increases beta-cell viability/functionality [20]. However, the islet-preserving effect of PAT could also stem from reduced glucotoxicity due to the competition for nutrients at co-culture conditions. In contrast, PAT removal increased beta-cell proliferation and the pancreatic *Gck*, *Glut2*, and *Pdx1* expression. We attribute these in vivo findings to compensatory beta-cell hyperplasia in response to HFD-induced systemic insulin resistance due to e.g., hepatic fat accumulation and inflammation [39].

In conclusion, additional studies are required to fully elucidate the role of PAT in the regulation of whole-body metabolism, pancreatic islets, and intrapancreatic adipogenesis. Nevertheless, our data suggest that PAT is a metabolically active fat depot with systemic metabolic effects far greater than suggested by its small size.

## Supplementary information

Supplemental material
