# Supplementary material for: Peripancreatic adipose tissue protects against high-fat-diet-induced hepatic steatosis and insulin resistance in mice
Source: Int J Obes (Lond). 2020 Aug 25;44(11):2323–34. doi: 10.1038/s41366-020-00657-6 (PMC7577900; doi:10.1038/s41366-020-00657-6)
Supplement: Supplementary file 1 — Supplemental material [file 41366_2020_657_MOESM1_ESM.pdf]

Supplementary Information

for

Peripancreatic adipose tissue protects against high fat diet-induced hepatic steatosis and insulin resistance in mice

***Running title*: Pancreatic fat protects against insulin resistance**

Belén Chanclón[1], Yanling Wu[1], Milica Vujičić[1], Marco Bauza-Thorbrügge[1], Elin Banke[1], Peter Micallef[1], Julia Kanerva[1], Björn Wilder[1], Patrik Rorsman[1,2], Ingrid Wernstedt Asterholm[1*]

[1]Department of Physiology (Metabolic Physiology), Institute of Neuroscience and Physiology, Sahlgrenska Academy, University of Gothenburg, SE405 30 Gothenburg, Sweden.

[2]Oxford Centre for Diabetes, Endocrinology and Metabolism, Radcliffe Department of Medicine, University of Oxford, Oxford, OX4 7LE, UK.

*Corresponding author: IWA@neuro.gu.se

List of contents

Supplementary method descriptions

Figure S1. Additional PAT and PAT adipocyte data.

Figure S2. Additional information from the HFD time course study.

Figure S3. Additional information from the PAT-ectomy study.

Figure S4. Effect of PAT-islet co-culture on insulin release

Table S1. Adipose depot weight in the HFD time course study**.**

Table S2. Pearson correlations.

Table S3. Real-time PCR primer sequences.

Supplementary method descriptions

Lipid and glucose uptake in vivo

In vivo tissue lipid and glucose uptake were assessed using respectively, [9,10-^{3} H(N)]-triolein (2 μCi/mouse, Perkin Elmer, Boston, MA, USA) and D-[U-^{14} C]-glucose (5 μCi/mouse, Perkin Elmer, Boston, MA, USA) tracers incorporated in a 20% intralipid emulsion (Sigma-Aldrich, St Louis, MO, USA). An oral load of the radioactive emulsion (300 μl/mouse) was given to 4-hour fasted adult male mice. After 2h, tissues were weighed and homogenized in 1 ml 2:1 chloroform: methanol and stored at 4°C overnight. $CaCl_2$ (0.5 ml, 1M) was added to all samples and the aqueous (oxidized fraction) and the organic phase (containing neutral lipids) were separated by centrifugation at 4°C and 3 000 rpm for 20min. Both phases were transferred to scintillation vials and 5ml of scintillation cocktail (Ultima Gold™, a high flash-point LSC-cocktail for counting aqueous and non-aqueous samples, Perkin Elmer, Waltham, Massachusetts, USA) was added. Incorporation of ^{3}H-triolein and ^{14}C-glucose was quantified in a beta-counter (Perkin Elmer, USA) and results are expressed as fraction (%) of total ^{3}H or ^{14}C counts per min (CPM) per μg tissue.

Lipogenesis and lipolysis assay

Primary white adipocytes from MWAT and PAT (pooled from 3-4 mice/sample) were isolated by collagenase digestion and kept in a Krebs Ringer buffer with 25 mM HEPES (KRH), 1% bovine serum albumin (BSA), 2mM glucose and 200 nM adenosine (pH 7.5). Cells were washed twice and diluted into a 1.5% cell suspension in a low glucose KRH buffer (3.5% BSA, 0.55 mM glucose and 200 nM adenosine; pH 7.5). Incorporation of ^{3}H-glucose into cellular lipids was measured in triplicates after 30min as previously described (26). For lipolysis measurements, glycerol released into media was measured with free glycerol assay according to manufacturer protocol (G7793 and F6428, Sigma-Aldrich).

Quantitative real-time PCR

RNA from GWAT, IWAT, MWAT, PAT, pancreas and liver was isolated using commercial kits (Qiagen, Germantown, MD, USA and ReliaPrep™ RNA Cell Miniprep System; Promega, Madison, WI, USA) and cDNA was prepared using High

capacity RNA-to-DNA kit (Life Technologies, Carlsbad, CA, USA) according to the manufacturer's instructions. Real-time PCR products were detected using SYBR Green (Life Technologies) and quantified using the relative Ct-method. Suitable reference genes (*Actb* and/or *Tbp*) were identified by the NormFinder algorithm using GenEx (MultiD) as described (27). Primer sequences are available in **Supplementary Table 3**.

Isolation of stromal vascular fraction (SVF) cells

Male mouse GWAT, IWAT and PAT were collected, minced (~1mm^3 pieces) and digested by shaking at 37°C in a buffer containing (mM) 123 NaCl, 5 KCl, 5 $CaCl_2$, 1.2 KH_2PO4, 1.2 $MgSO_4$, 25 HEPES and 2 glucose, supplemented with 200 nM adenosine, 1.5% BSA and 2 mg/ml collagenase type II. The digest was filtered through 100-µm mesh and centrifuged for 5 min at 500g. Red blood cells were removed with RBC lysis buffer (Biolegend, San Diego, CA, USA) treatment for 2 min at room temperature. Cells were washed, resuspended in DMEM 10% FBS and counted on automatic cell counter (Countess 2, Thermo Fisher, Waltham, MA, USA) after which they were stained for flow cytometry analysis. PAT samples were pooled from 20 mice to a total n=5 due to their small size.

Flow cytometry

Isolated SVF cells (0.5x10^6 cells per sample) were washed twice in PBS. Non-specific binding was blocked by incubating cells with anti-mouse CD16/CD32 antibody (Miltenyi biotech, Bergisch Gladbach, Germany) in PBS 1% BSA for 15 min in dark on ice. Fluorescently conjugated antibodies were added for 1h in dark on +4°C in appropriate combinations: anti-mouse CD45 APC-Cy7 (BioLegend), anti-mouse F4/80 PE-Cy7, anti-mouse CD3 FITC, anti-mouse CD140 APC (all Miltenyi), anti-mouse CD4 FITC, anti-mouse CD8 APC, anti-mouse CD19 PerCP-Cyanine 5.5, anti-mouse NK1.1 PE-Cy7, anti-mouse CD11b PerCP-Cyanine 5.5, anti-mouse CD11c PE (all eBioscience, San Diego, CA, USA). After incubation, cells were washed twice and resuspended in PBS 1% BSA. Dapi (Miltenyi) was added as a dead cell marker. Cells were acquired on FACSCANTO 2 flow cytometer (BD Bioscience, Franklin Lakes, NJ, USA). 20 000 events per sample were recorded. All fluorophores were compensated with compensation controls, and gates were set with Fluorescence Minus One (FMO) controls. Cells were gated based on their FSC-SSC appearance, followed by selection

of single live $CD45^+$ (leukocytes) or $CD45^-$ (fibroblasts) cells. The flow cytometry data was analysed using FlowJo software version 10.6.0 (FlowJo, LLC, Ashland, OR).

Perilipin-1 staining of paraffin-embedded sections

Paraffin-embedded sections were rehydrated and antigen retrieval was performed by heating samples for 20 min with 10 mM citric acid 0.05% Tween (pH=6). Non-specific staining was blocked with 3% donkey serum in PBS for 30 min on RT. Sections were incubated with anti-Perilipin-1 antibody (1:150, Abcam, Cambridge, UK) diluted in incubation buffer (PBS, 1% BSA; 1% donkey serum, 0.3% Triton-X) overnight in dark at +4°C. After wash (3x10 min in PBS), secondary antibody (donkey anti-rabbit Alexa Fluor 555, 1:200, Thermo Fisher) was added for 40 min on RT in dark. After final wash, slides were mounted with Vectashield antifade mounting medium with Dapi (Vector laboratories, Burlingame, CA, USA). Sections were visualised on LSM 700 laser scanning confocal microscope (Zeiss, Oberkochen, Germany). Dapi was excited with 405 nm laser and Alexa Fluor 555 with 555 nm laser. Image acquisition was performed with Zeiss Zen Black 2012 software. Positive and negative controls were included in the experiment.

Oral glucose tolerance test (OGTT), glucose, insulin and glucagon measurements

OGTT was performed in PAT-ectomized and control mice as indicated in **Supplementary Fig. 3**. Mice were fasted for 4 hours and blood samples were taken from the tail vein. Blood samples were collected just before and 15, 30, 60 and 120min after an oral load of D-glucose (2.5 g/kg, Sigma Aldrich, St. Louis, MO, USA) dissolved in water. Blood glucose levels were measured using a glucometer (Contour from Bayer; Baser, Switzerland). Serum insulin and glucagon levels were measured by commercial ELISA kits (Mouse Insulin and Glucagon ELISAs, Mercodia, Uppsala, Sweden).

Adipocyte, hepatic lipid droplet, pancreatic islet and intrapancreatic adipocyte size measurements

GWAT, IWAT, MWAT, PAT, liver and pancreas samples were fixed for 72h in 4% phosphate-buffered formalin solution (VWR Chemicals, Stockholm, Sweden), stored in 50% ethanol and embedded in paraffin. Paraffin sections (four 7μm sections/mouse

and 4-5 mice/group for adipose tissues and liver & two 7μm sections/mouse, 17-38 islets/section, and 3-4 mice/groups for pancreas) were stained with hematoxylin&eosin (H&E) solution. Five 20x images per animal were obtained using a light microscope (Olympus BX60 & PlanApo, 20x/0.7, Olympus, Tokyo, Japan). Adipocyte area, hepatic lipid droplet area and number, islet area and number, and intrapancreatic adipocyte area and number were measured using ImageJ v1.47 software (National Institutes of Health, Bethesda, MD, USA) as previously described (25). Average adipocyte and islet size were presented as μm^2, and liver lipid droplet size distribution was presented as % of total. The investigator was blinded to the group allocation.

Tissue lipid content

Frozen tissue (10-30 mg of pancreas; 30-100 mg of liver) was washed in cold PBS before homogenization in 5% IGEPAL/ddH_2O using a tissue lyzer (30s at 25Hz). Samples were slowly heated to 85°C for 2-5min and then cooled down to room temperature. They were centrifuged at maximum speed to remove insoluble material before proceeding with the triglyceride content assay following instructions of manufacturer (Randox Laboratories, Dublin, United Kingdom).

Islet and PAT co-culture

Pancreatic islets (12 per well) were put on top of the 24-mm Transwell membrane inserts (Costar, Washington DC, USA) and co-cultured with finely minced (~1mm^3 pieces) PAT explants (~10 mg/sample, bottom well) in DMEM at 11 mM glucose overnight at 37°C, 5% CO_2. After incubation, medium was collected for insulin measurements and pancreatic islets were collected for glucose-stimulated insulin secretion assay.

Glucose-stimulated insulin secretion (GSIS)

Pancreatic islets were washed in Krebs buffer 0.1% BSA with no glucose, before placing them in individual tubes (N=12 islets/tube). In order to measure GSIS, islets were incubated with Krebs buffer 2.8 mM or 16.8 mM glucose in an orbital-shaker incubator at 80-100 rpm during 1h at 37°C. The supernatant was collected and stored at -80°C and islets were homogenized in HCl and frozen pending later measurements of insulin.

Figure S1. Maximal adipocyte size of PAT, MWAT, IWAT and GWAT in (**A**) chow diet-fed mice (n=7-10 mice) and (**B**) 8-week HFD-fed mice (n=4-5 mice). *In vitro* (**C**) lipogenesis and (**D**) lipolysis in isolated PAT and MWAT adipocytes from chow and 8-weeks HFD-fed mice (n=3/group, each group contains adipocytes from 3-4 mice). (**E**) Flow cytometry analysis (n=5-6/group) of SVF isolated from PAT, GWAT and IWAT (fibroblasts (CD45$^-$PDGFRa$^+$), macrophages (F4/80$^+$CD11b$^+$), dendritic cells (CD11b$^+$CD11c$^+$) and different types of lymphocytes (CD3$^+$, CD8$^+$, CD19$^+$, CD4$^+$, NK1.1$^+$, CD4$^+$NK1.1$^+$). All values are expressed as mean ± SEM. * p<0.05, ** p<0.01 and *** p<0.001. IWAT; inguinal adipose tissue, MWAT; mesenteric adipose tissue and GWAT; gonadal adipose tissue.

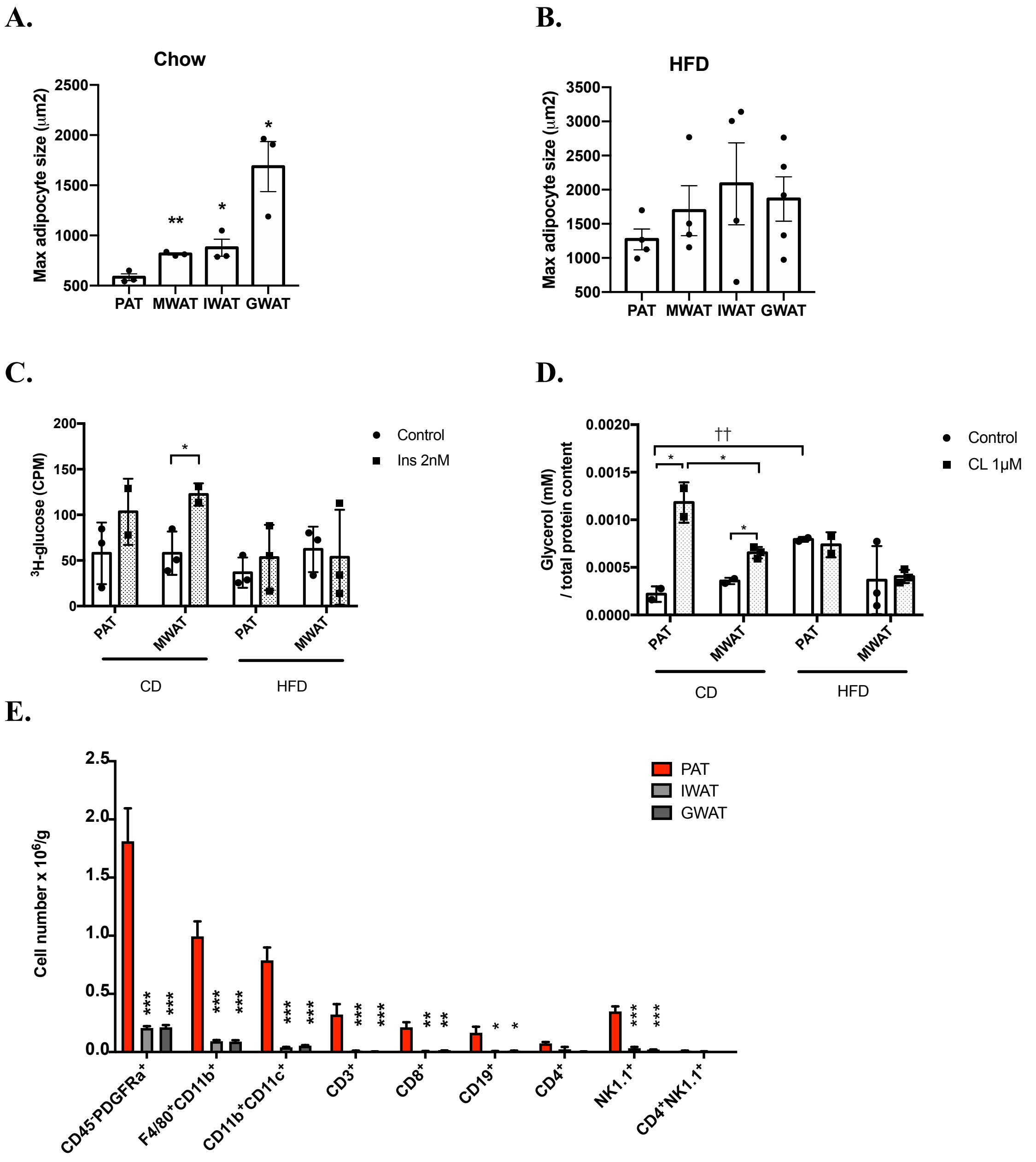

Figure S2. (**A**) Schematic experimental design of the HFD time course study and the (**B**) body weights of the mice used in this study. All values are expressed as mean ± SEM. * p<0.05, ** p<0.01 and *** p<0.001 for high-fat vs chow diet.

A.

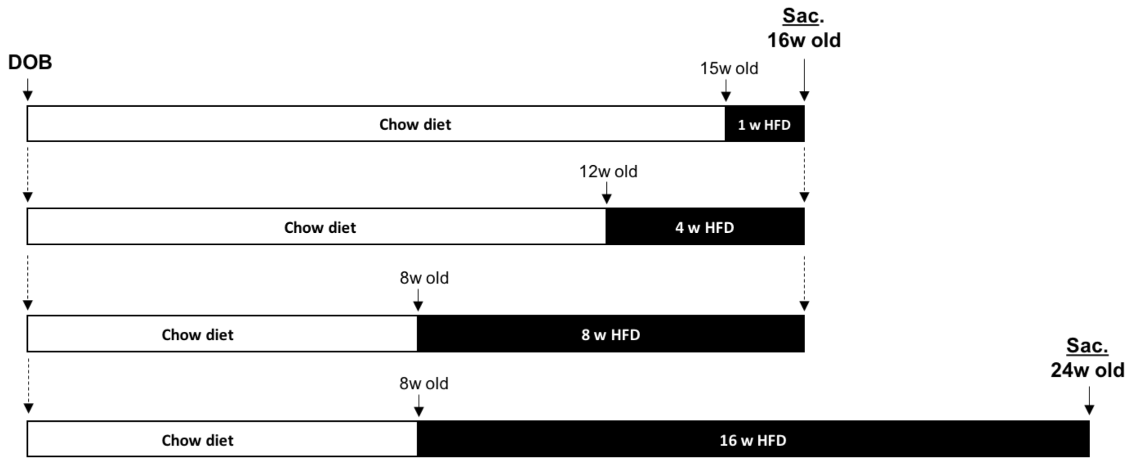

B.

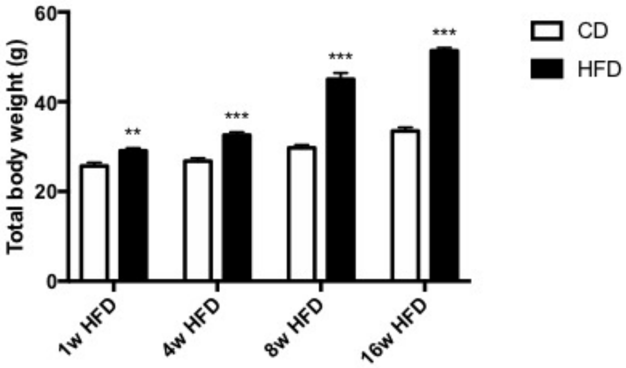

Figure S3. (A) Schematic experimental design of PAT-ectomy studies. **(B)** PAT adipocyte size distribution in PAT-ectomized mice after 16-week HFD-feeding. **(C)** Fat depots weight and (**D**) % of ballooning area in PAT-ectomized mice after 16-week HFD-feeding. All values are expressed as mean ± SEM. *** p<0.001. IWAT; inguinal adipose tissue, MWAT; mesenteric adipose tissue and GWAT; gonadal adipose tissue.

A.

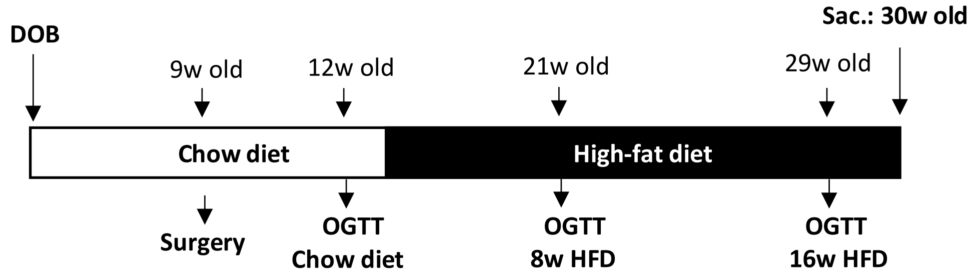

B.

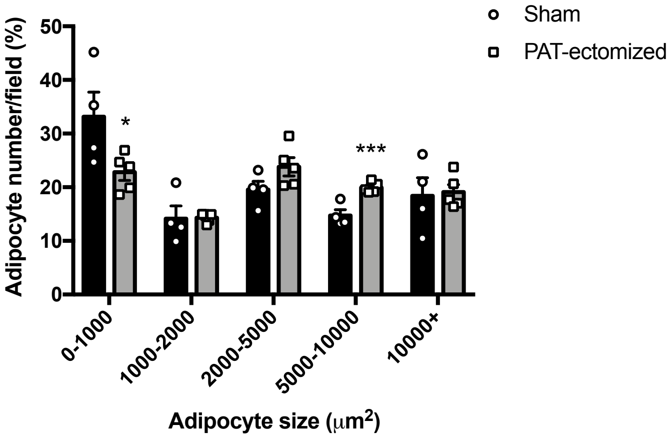

C.

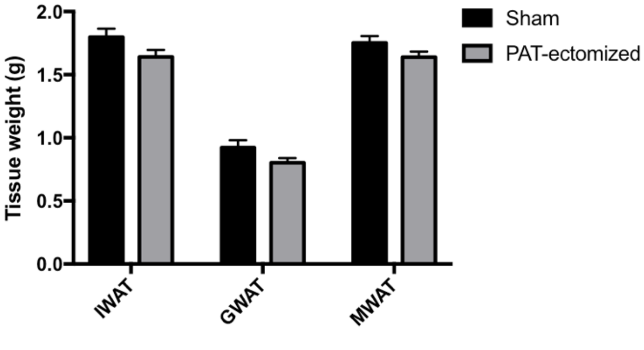

D.

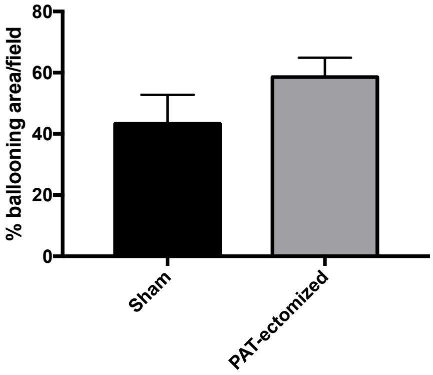

Figure S4. (**A**) Insulin secretion from islets during the 18 h co-culture time (n=8 + 16). (**B**) Glucose-stimulated insulin secretion in fresh islets (left panel), and control and PAT co-cultured islets (right panel), n=3-8. All values are expressed as mean ± SEM. * p<0.05 and *** p<0.001 for the indicated comparisons.

A.

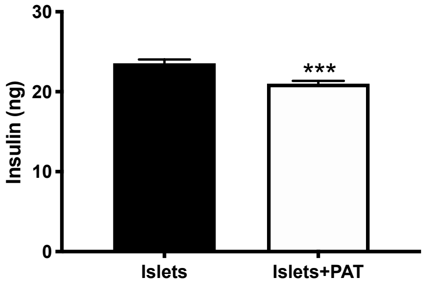

B.

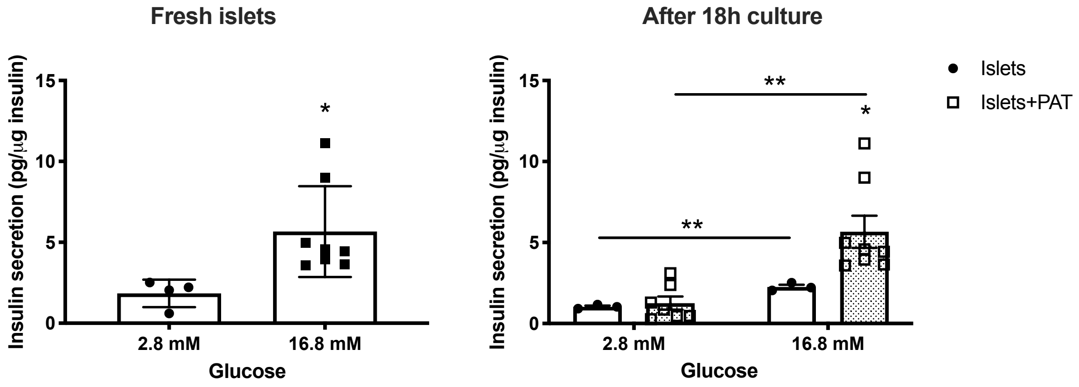

Table S1. Adipose depot weight in the HFD time course study. * p<0.05, ** p<0.01 and *** p<0.001 for the difference between chow and HFD (n=6-10/group). PAT; peripancreatic adipose tissue, IWAT; inguinal adipose tissue, GWAT; gonadal adipose tissue and MWAT; mesenteric adipose tissue.

		PAT (mg)	IWAT (mg)	GWAT (mg)	MWAT (mg)
**1-week**	**Chow**	8 ± 1	138 ± 7	205 ± 9	207 ± 19
	**HFD**	14 ± 3*	329 ± 38***	410 ± 27***	513 ± 49***
**4-week**	**Chow**	11 ± 1	168 ± 4	253 ± 16	264 ± 12
	**HFD**	14 ± 3***	329 ± 38***	410 ± 27***	513 ± 49***
**8-week**	**Chow**	14 ± 2	159 ± 19	296 ± 22	280 ± 34
	**HFD**	64 ± 7***	935 ± 89***	1309 ± 66***	1224 ± 112***
**16-week**	**Chow**	29 ± 3	345 ± 33	555 ± 57	639 ± 54
	**HFD**	129 ± 31**	1542 ± 54***	905 ± 55***	1736 ± 48***

Table S2. Pearson correlations. A p-value < 0.05 indicates that the Pearson's correlations coefficient (R) is significantly different from zero. PAT; peripancreatic adipose tissue, IWAT; inguinal adipose tissue, GWAT; gonadal adipose tissue and MWAT; mesenteric adipose tissue.

**Chow (N=10)**		**PAT**	**IWAT**	**GWAT**	**MWAT**	**Liver**
**Body weight**	***R***	.883	.879	.909	.900	.654
	p-value	.001	.001	<.001	.001	.040
**Liver**	***R***	.430	.780	.870	.691	1
	p-value	.215	.008	.001	.039	

**16 weeks HFD (N=15)**		**PAT**	**IWAT**	**GWAT**	**MWAT**	**Liver**
**Body weight**	***R***	.446	.789	-.339	.019	.608*
	p-value	.096	<.001	.216	.947	.016
**Liver**	***R***	.649**	.253	-.648	-.134	1
	p-value	.009	.362	.009	.633	

Table S3. Primer sequences used for quantitative real-time PCR.

Adiponectin	TGTTCCTCTTAATCCTGCCCA	CCAACCTGCACAAGTTCCCTT
Arg1	CTCCAAGCCAAAGTCCTTAGAG	AGGAGCTGTCATTAGGGACATC
Atp6	ACTTGCCCACTTCCTTCCACA	TAAGCCGGACTGCTAATGCCA
Beta-actin	GACCCAGATCATGTTTGAGA	GAGCATAGCCCTCGTAGAT
B3ar	GGCCCTCTCTAGTTCCCAG	TAGCCATCAAACCTGTTGAGC
Cd206	TGTGGTGAGCTGAAAGGTGA	CAGGTGTGGGCTCAGGTAGT
Chrebp	GCATCCTCATCCGACCTTTA	GATGCTTGTGGAAGTGCTGA
Cpt1a	TGGTGGGAGGAATACATC	CAGAAGACGAATAGGTTTGAG
Fasn	GGAGGTGGTGATAGCCGGTAT	TGGGTAATCCATAGAGCCCAG
F4/80	CTTTGGCTATGGGCTTCCAGTC	GCAAGGAGGACAGAGTTTATCGTG
Gck	GGGAACAACATCGTGGGACT	CCTTCCACCAGCTCCACATT
Glut2	GTCTCTGTGCTGCTTGTGGA	GGACCTGGCCCAATCTCAAA
Glut4	GTAACTTCATTGTCGGCATGG	AGCTGAGATCTGGTCAAACG
Gpr40	TTTCATAAACCCGGACCTAGGA	CCAGTGACCAGTGGGTTGAGT
Il1b	AATGAAAGACGGCACACCCA	TGCTTGTGAGGTGCTGATGT
Insr	TCATGGATGGAGGCTATCTGG	CCTTGAGCAGGTTGACGATTT
Ins1	TAATGGGCCAAACAGCAAAG	GGGTAGGAAGTGCACCAACA
Ins2	TCAAAAACCATCAGCAAGCA	ACCAGGTGGGAACCACAAA
Leptin	GTCCAGGATGACACCAAAACC	GACAAACTCAGAATGGGGTGAA
Mcp1	ACTGAAGCCAGCTCTCTCTTCC	TTCCTTCTTGGGGTCAGCACAG
Pdx1	CCATGAACAGTGAGGAGCAGT	GTGATCCCAGCGAGCTTGTA
Pepck	CATGAAAGGCCGCACCA	TCATGATCCGCATGCTGG
Pparg	TTGAGTGCCGAGTCTGTGG	GGCATTGTGAGACATCCCCA
Saa1	ACACCAGGATGAAGCTACTCACCA	CCCTTGGAAAGCCTCGTGAACAAA
Saa2	AGCTGGCTGGAAAGATGGAGACAA	TGTCCTCTGCCGAAGAATTCCTGA
Saa3	TAAAGTCATCAGCGATTCCAGAG	CAACCCAGTAGTTGCTCCTCTTC
Scd1	TTCTTGCGATACACTCTGGTGC	CGGGATTGAATGTTCTTGTCGT
Srebp1c	GGGCACTGAAGCAAAGCTGA	CTGGTTGCTGTGCTGCAAGA
Tbp	AGAACAATCCAGACTAGCAGCA	GGGAACTTCACATCACAGCTC
Tnfa	ATGGCCTCCCTCTCATCAGT	GCAGCCTTGTCCCTTGAAGA
Ucp1	GTGAAGGTCAGAATGCAAGC	AGGGCCCCCTTCATGAGGTC
